# Yeast Augmented Network Analysis (YANA): a new systems approach to identify therapeutic targets for human genetic diseases

**DOI:** 10.12688/f1000research.4188.1

**Published:** 2014-06-02

**Authors:** David J. Wiley, Ilona Juan, Hao Le, Xiaodong Cai, Lisa Baumbach, Christine Beattie, Gennaro D'Urso

**Affiliations:** 1 Department of Molecular and Cellular Pharmacology, University of Miami Miller School of Medicine, Miami, FL, 33136, USA; 2 Department of Neuroscience, Ohio State University, Columbus, OH, 43210, USA; 3 Department of Engineering, University of Miami, Miami, FL, 33124, USA; 4 Integrated Functional Cancer Genomics, TGEN, Phoenix, AZ, 85004, USA

## Abstract

Genetic interaction networks that underlie most human diseases are highly complex and poorly defined. Better-defined networks will allow identification of a greater number of therapeutic targets.

Here we introduce our Yeast Augmented Network Analysis (YANA) approach and test it with the X-linked spinal muscular atrophy (SMA) disease gene *UBA1. *First, we express *UBA1* and a mutant variant in fission yeast and use high-throughput methods to identify fission yeast genetic modifiers of *UBA1*. Second, we analyze available protein-protein interaction network databases in both fission yeast and human to construct *UBA1* genetic networks. Third, from these networks we identified potential therapeutic targets for SMA. Finally, we validate one of these targets in a vertebrate (zebrafish) SMA model. This study demonstrates the power of combining synthetic and chemical genetics with a simple model system to identify human disease gene networks that can be exploited for treating human diseases.

## Introduction

Many disease-associated genes have been identified, yet most genetic diseases remain untreatable. One path to treatment is to develop extensive genetic networks in which human disease genes function (or dysfunction) and then target therapies to the genes identified in those networks. Eventually, an individual’s own genotype for proteins in the network may also be considered in the therapeutic options. Developing gene networks is already widely recognized as a powerful approach to identify new drug targets ^1– 6^. Gene networks are based on the principle that networks contain proteins that interact (physically and/or functionally) and that these interactions govern most, if not all, cellular functions. Importantly, gene network interactions are often conserved in different organisms even though the output of the networks may differ ^7^. Candidate networks can be generated from studies in model organisms and then extrapolated to human cells. One can expect that, for disease gene networks, some of the interacting genes might modulate the disease phenotype thus representing potential therapeutic targets.

The direct identification of disease networks from studies in human cells is, of course, challenging because of the large number of potential proteins and incomplete knowledge of how the activity of any one protein may affect the network output. Analysis of networks would benefit from studies in a tractable model organism amenable to high throughput methods. Given that network interactions may be conserved between humans and model organisms, even while the outputs may differ, we hypothesized that we can identify human disease networks in the fission yeast *Schizosaccharomyces pombe*, a simple, genetically tractable model organism, coupled to existing protein-protein interaction databases. We can then transfer that network knowledge to human cells with the goal of identifying therapeutic target genes.

How valid is the approach of using fission yeast networks to identify human disease gene networks? First, the proteins that control most core cellular functions are in fact evolutionarily conserved, underscoring the “deep homology” that exists between all living organisms ^8, 9^. For example, in *S. pombe* >65% of the genome is orthologous to human ( http://orthomcl.org/orthomcl/ and http://www.pombase.org). Second, many of the cellular processes implicated in human disease, e.g. vesicular transport, protein folding, metabolism, and RNA processing, are also evolutionarily conserved and highly interconnected ^10^. Importantly, the value of a simple model organism for discovering “druggable” genetic pathways has recently been demonstrated for the budding yeast *Saccharomyces cerevisiae* ^11^, and thus extending the study of human disease genes to fission yeast seems promising.

**Figure 1.**
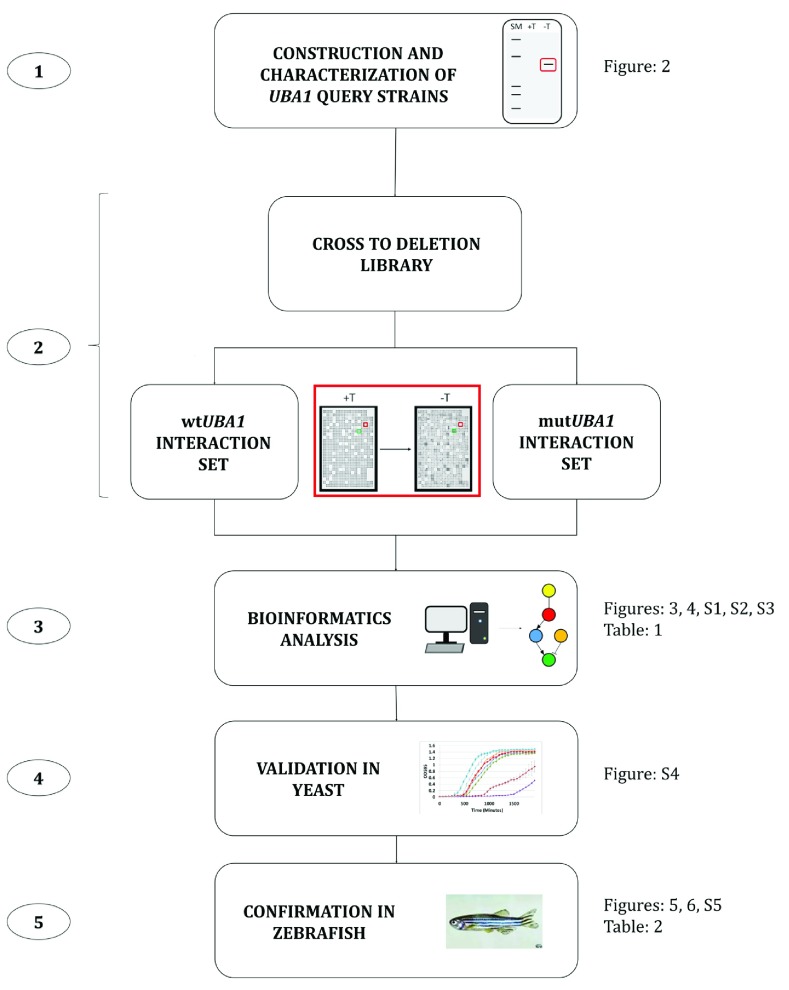
YANA analysis of *UBA1* genes. Numbers illustrate various steps in our analysis. (1) Construction of query strains by integrating wildtype and mutant variants of the *UBA1* gene into *pombe*. (2) Crossing the query strains (mutant or wildtype) to a complete non-essential deletion collection. (3) Bioinformatic analysis to generate the top ‘gene hits’. (4) Validation of the top hits in yeast. (5) Confirmation of the top hits in zebrafish.

Our approach ( Figure 1) involves the expression of human disease associated genes in *S. pombe* and then the analysis of their effect on yeast fitness (growth). We performed high-throughput synthetic genetic array (SGA) screens to identify the fission yeast genetic modifiers that alter this effect, as measured by a simple yeast growth assay. The genetic modifiers are then assembled into human disease gene networks or clusters (using protein-protein interaction datasets from both *S. pombe* and humans). Any modifiers or genes in the networks or clusters represent potential therapeutic targets. This unbiased high-throughput approach could be widely and rapidly applied to many different disease-associated genes at relatively low cost.

To demonstrate the power of YANA, we considered spinal muscular atrophy, a common neurodegenerative disease affecting approximately 1/6000 births worldwide and the number one genetic cause of infantile death in the United States ^12^. Most cases (>90%) are caused by deletion of *SMN1*, a gene encoding the survival motor neuron (SMN), a protein involved in the assembly of spliceosomal small nuclear ribonuceoproteins (snRNPs) ^13^. In contrast, X-linked spinal muscular atrophy is caused by mutations in *UBA1*, a gene encoding the ubiquitin activating (UBA) enzyme 1, an E1 ubiquitin ligase ^14, 15^. Consistent with a role of ubiquitination in spinal muscular atrophy and SMN biology, it was recently shown that ubiquitin-mediated proteolysis regulates SMN stability ^16, 17^. Therefore, we systematically investigated the genetic network for *UBA1* to better understand the potential connections between UBA1, its modifiers and the spinal muscular atrophy phenotype. Using YANA we identified several potential therapeutic targets and validated one of these targets in a SMA vertebrate (zebrafish) model.

## Methods

### Query Strain Creation

UBA1 was cloned into a pENTR/D-TOPO vector (Life Technologies, Cat # K2400-20) from cDNA (Origene, Cat. # SC320329) following the manufacturer’s protocols. The following primers were used: forward primer: 5′- CACCATGTCCAGCTCGCCGC-3′; reverse primer: 5′- TCAGCGGATGGTGTATCGGAC-3′. Genetic insertion was confirmed by sequencing ( http://sylvester.org/shared-resources/oncogenomics). UBA1 (G1617T), mut *UBA1*, was created by site directed mutagenesis using a QuikChange Lightning Site-Directed Mutagenesis kit (Agilent Technologies, Cat. # 210518; the detailed protocol is available in the kit). The primers used were: forward primer: 5′-GCAGCTGTGCGCCAAATTAATCCACATATCCGG-3′; reverse primer: 5′-CCGGATATGTGGATTAATTTGGCGCACAGCTGC-3′. LR Gateway reactions (Gateway Cloning Protocols: http://www.lifetechnologies.com/us/en/home/life-science/cloning/gateway-cloning/protocols.html#lr) were then performed to flip the *UBA1* genes into destination vectors, to create N-terminal HA-tagged UBA1 under the control of the *nmt1* promoter (LR Clonase II from Life Technologies, Cat # 11791020). The newly generated expression vectors were then integrated into an *h ^-^ leu1-32 ura4-D18 Ade6-M210 S. pombe* strain (PN572) to create a wt *UBA1* query strain ( *h ^-^ integrated pjk148-nmt1 ^3X^-HA-UBA1-nmt1 ^term.^ leu1-32 ura4-D18 Ade6-M210*) and a mut *UBA1* query strain ( *h ^-^ integrated pjk148-nmt1 ^3X^-HA-UBA1(G1617T)-nmt1 ^term.^ leu1-32 ura4-D18 Ade6-M210*). All media, growth conditions, and genetic manipulations were as previously described ^18^.

### Western Blot and Growth Curves

Strains containing wt *UBA1* and mut *UBA1* were grown exponentially in PMG media (Sunrise Scientific Cat. #2060, keeping the OD _595_ below 0.4) or eight generations at 32°C and then induced for expression of the HA-tagged wt *UBA1* and mut *UBA1* by washing the cells three times with sterile water to remove thiamine. Cells were then grown exponentially for 16 hours and then lysed using a FastPrep 120 bead beater (MP Biomedical), followed by boiling in sample buffer (2x Laemmli Sample Buffer, Bio-Rad Cat. #161-0737) and then clarified by centrifugation. The expression of UBA1 was confirmed by Western Blot analysis using an anti-HA antibody at 1:2000 dilution (Covance, Cat #MMS-101P, AB_10063488) and standard procedures ^19^. Growth curve analysis was completed in the presence and absence of thiamine (final concentration, 15 µM; Sigma, Cat. #T4625). Removal of thiamine was achieved by washing the cells three times with sterile water. Cells were incubated in the absence of thiamine for 22 hours under exponential growth, diluted and 120 µl added to the Tecan Infinity F200 plate readers (starting OD _595_ of 0.05) for growth curve analysis with an n=5 for each sample. For validation experiments, *cul3* and *gsk3* were not pre-induced, while *pub1* was pre-induced for 22 hours. The deletions of *cul3*, *pub1*, and *gsk3* were confirmed by PCR analysis of genomic DNA from each strain, as previously described ^20^.

### SGA screening

Query strains were grown in liquid media and then pinned to agar in a 384-format using a RoToR HDA (Singer Instruments). The query was then crossed to the *S. pombe* haploid deletion library (Bioneer, Version 3.0 equivalent) on SPAS media (details can be found at http://www-bcf.usc.edu/~forsburg/media.html) using a modified SGA procedure ^21^. For germination, four replicates of each cross were pinned to a 1536 format on selective PMG media containing thiamine, adenine (225 mg/L, Sigma Cat. #A8751), leucine (225 mg/L, Sigma Cat. #L8912), and the antibiotic G418 (150 µM, Gold Bio Cat. #G-1418) (PAUT+G418). After three days, the plates were then pinned to both PAUT+G418 (non-inducing) and PAU+G418 (inducing) plates. The colonies growing on the PAUT+G418 plates were documented on a flatbed scanner for the next three consecutive days. The PAU+G418 plates were grown for two days, and then re-pinned to fresh PAU+G418 plates and documented over the next three consecutive days. Based on the growth characteristics of the wt *UBA1* strain, the plates were then pinned to fresh PAU+G418 plates and documented for an additional three days.

### Hit analysis

The documented plates were analyzed for ‘Hits’ representing a growth defect (SL) or growth suppressor (SS) using ScreenMill software ^22^. The software is used to quantify colony size for each individual cross and then to normalize the quantified plates with and without replicate exclusion for each quadruplicate of the query crossed to a specific deletion strain. Data was then compared (between non-induced versus induced) and ranked in Excel (P≤0.05) (Microsoft). The orthologs were then identified based on curated data from PomBase ( www.pombase.org; build 2013-11-11-v1), OrthoMCL ( http://orthomcl.org/orthomcl/; Version 5), InParanoid8 ( http://inparanoid.sbc.su.se/cgi-bin/index.cgi; Version 8.0), and Homologene ( http://www.ncbi.nlm.nih.gov/homologene; build 67).

### Bio-Informatics

The *S. pombe* and human ‘Hits’ were analyzed in String ( www.string-db.org) to map protein-protein interactions limited to data from experiments at the highest confidence level (0.900), and named *pombe* primary and human primary. The *S*. *pombe* primary was then extended by adding first degree neighbors to the original *pombe* ‘Hits’, keeping all interactions based on data from experiments at the highest confidence level (0.900). From the extended *pombe* network interaction map, new ‘Hits’ were extracted that corresponded to at least two previous ‘orphans’ (‘Hits’ that were not previously mapped) that interact through a nearest neighbor. All ‘Hits’ were then analyzed through PubMed ( http://www.ncbi.nlm.nih.gov/pubmed/) for relevance in terms of SMA, with special focus on the ‘Hits’ in the interaction maps.

### Morpholino (MO) injection and drug test

Transgenic *Tg(mnx1:0.6hsp70:GFP)os26* embryos that express GFP in ventrally projecting motor axons, referred to as *Tg(mnx1:GFP)* embryos, were used for all zebrafish experiments. Embryos were staged according to Fritz *et al.* ^23^ All fish were grown and maintained in the Ohio State University (OSU) zebrafish facility following established protocols and OSU animal welfare guidelines as stated in Dr. Beattie's animal protocol (On file at OSU: 2009A0141-R1). *Tg(mnx1:GFP)* embryos were injected with 4 ng *smn* MO at the one- to 2-cell stage to knock down Smn as previously described ^24^. At 10 hours post-fertilization (hpf) injected embryos (in their chorions) were placed in Petri dishes in fish water (60 µg/ml Instant Ocean ^®^ sea salts) containing compounds in 0.75% dimethyl sulfoxide (DMSO) or 0.75% DMSO only and incubated at 28.5°C in incubator until 28 hpf.

*Tg(mnx1:GFP)* embryos at 28 hpf were anesthetized with tricaine (250 µg/ml, Sigma A-5040) and fixed overnight at 4°C in 4% formaldehyde/PBS. After removing embryos from fix, they were mounted on glass coverslips for observation under a Zeiss Axioplan microscope. Motor axons innervating the mid-trunk (myotomes 6–15) on both sides of the fish were scored as described ^25^.

The control *topped ^b458^* embryos were treated and fixed as described above for *Tg(mnx1:GFP)* embryos; the *topped* mutants, however, were processed for znp1 antibody (Hybridoma Bank Cat#znp-1, AB_531910) labeling as previously described ^26^ to visualize motor axons for scoring as they did not have the *Tg(mnx1:GFP)* in the background.

### Zebrafish western blot

Samples of zebrafish embryos injected with 4 ng *smn* MO at the one- to two-cell stage and uninjected embryos were treated from 10 to 28 hpf with DMSO or with DMSO and compounds as described above were collected for western blot.

Samples were generated by boiling 25 identically treated embryos in 75 µl blending buffer (63 mM Tris (pH 6.8), 5 mM EDTA, 10% SDS). 10 µl (equivalent to 3 embryos or 75 µg of protein) were added to 10 µl of sample buffer (100 mM Tris (pH 6.8), 0.2% bromophenol blue, 20% glycerol, 200 mM dithiothreitol) and run on a 10% polyacrylamide gel, blotted to nitrocellulose, probed with mouse anti-Smn (1/500; MANSMA12, a gift from Dr. G.E. Morris or anti HuD (1/1000; Santa Cruz Cat#sc-28299, AB_627765) and detected by chemiluminescence of bound HRP-conjugated mouse antibody. Blots were stripped and re-probed with mouse anti-β-actin (1/1000; Santa Cruz Cat #sc-47778, AB_626632).

## Results

**Figure 2.**
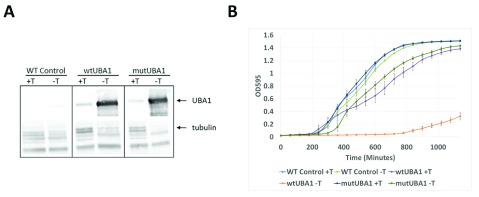
Construction of *S. pombe* strains integrated with human *UBA1*. Wildtype and mutant forms of *UBA1* were integrated into fission yeast under the control of the *nmt1* thiamine-repressible promoter. ( **A**) Western blot analysis of protein expression levels shows that both forms of *UBA1* (wildtype and mutant) are expressed to equivalent levels. ( **B**) Expression of wild-type *UBA1* inhibits *S. pombe* cell growth.

We expressed the human wildtype *UBA1* (wt *UBA1*) and the disease-causing variant 1617 *UBA1* (mutUBA1) in *S. pombe* under the control of a regulatable *nmt1* promoter ^27^. We identified strains with stable integrated human *UBA1* genes expressing equivalent levels of UBA1 proteins ( Figure 2A). Under non-inducing conditions (+ thiamine) all *UBA1*-containing strains had a similar growth rate to the control non- *UBA1*-containing wildtype strain ( Figure 2B). We then found that cells expressing human wt *UBA1* (fission yeast cells grown in media lacking thiamine to induce *UBA1* expression), experienced a decrease in cell growth. No growth defect was observed for yeast cells expressing mut *UBA1* when compared to the control ( Figure 2B).

We set out to identify yeast gene mutations that either enhance or suppress cell growth dependent upon expression of either wt or mut *UBA1* (i.e., epistatic modifiers). We used automated genetics to introduce the wt and mut *UBA1* genes into a deletion strain collection corresponding to more than 90% of the non-essential genes in *S. pombe* (ca. 80% of the complete genome). We then tested each of the >7000 unique strains expressing either wt or mut *UBA1* combined with a specific gene deletion, for growth properties. Each strain was scored for significance of growth difference compared to a control, and the top hits (P<0.05) were assembled into a database ( Table 1). The categories of growth phenotypes were those that were synthetic lethal for wildtype or mutant (wt-SL, or mut-SL) or synthetically suppressed for wildtype or mutant (wt-SS, or mut-SS). We identified 173 UBA1 or mutUBA1 modifiers. Notably, 145 of the modifiers (83.8%) were orthologous to human genes, and thus are potential drug targets to modify the SMA phenotype.

**Table 1.** *S. pombe* SGA top hits. The tops hits for wtUBA1 SL, wtUBA1 SS, mutUBA1 SL, and mutUBA1 SS were identified by bioinformatic analysis as described in the materials and methods section.

	wtUBA1 SL	wtUBA1 SS	mutUBA1 SL	mutUBA1 SS
1	SPAC24H6.03	SPBC365.06	SPBC8D2.01	SPAC11G7.02
2	SPAC1687.15	SPAC11G7.02	SPBC6B1.10	SPCC13B11.01
3	SPAC3G9.07c	SPCC31H12.05c	SPBC216.05	SPBC2G2.03c
4	SPBC6B1.10	SPBC31F10.13c	SPCC1795.01c	SPBC31F10.13c
5	SPAPYUG7.04c	SPCC1620.14c	SPBC24C6.11	SPCC1620.14c
6	SPBC24C6.11	SPAC57A10.02	SPBC1734.08	SPAC3G6.02
7	SPBC1734.08	SPAC1556.01c	SPAC9.13c	SPAC1556.01c
8	SPAC23D3.09	SPAC30D11.13	SPAC23D3.09	SPAC821.05
9	SPAC17A5.11	SPCC970.07c	SPAC57A10.02	SPBC6B1.04
10	SPAC16A10.05c	SPAC23H4.12	SPAC16A10.05c	SPCC1919.15
11	SPCP1E11.07c	SPCC11E10.08	SPCC895.07	SPBC23E6.09
12	SPBC146.13c	SPBC21D10.12	SPCP31B10.05	SPBC21D10.11c
13	SPAC821.09	SPCC1672.06c	SPCC550.12	SPAC22H10.07
14	SPBC23E6.09	SPAC3C7.03c	SPAC23H4.12	SPCC338.08
15	SPCC1682.16	SPBC32F12.02	SPBC11C11.02	SPBP35G2.08c
16	SPBC106.01	SPCC736.04c	SPBC1289.11	SPBC29A3.14c
17	SPCC622.16c	SPBC30D10.13c	SPCC126.02c	SPAC29A4.18
18	SPAC22H10.07	SPCC737.09c	SPBC13G1.13	SPBC1921.03c
19	SPBC13G1.13	SPBC56F2.01	SPCC188.13c	SPBC2F12.11c
20	SPBC16H5.06	SPBC2F12.11c	SPCC663.12	SPAPB1E7.02c
21	SPBC29A10.05	SPBC16A3.07c	SPCC338.05c	SPAC17A5.16
22	SPBC28F2.07	SPAC5D6.05	SPAC343.11c	SPAC20H4.03c
23	SPCC1827.08c	SPAC18G6.02c	SPCC1919.03c	SPCC1259.03
24	SPAC9G1.10c	SPAC19D5.01	SPAC343.18	SPAC18G6.02c
25	SPBC83.03c	SPAC688.11	SPCC737.09c	SPAPB1E7.06c
26	SPBC342.04	SPAC227.07c	SPBC106.16	SPAC1851.04c
27	SPCC4G3.19	SPAC1851.04c	SPAC3A11.14c	SPAC13G7.06
28	SPAC9G1.05	SPBC16H5.07c	SPAC589.02c	SPBC725.11c
29	SPCC4E9.02	SPAC17A5.18c	SPCC1450.05c	SPCC1739.12
30	SPCC794.11c	SPBC725.11c	SPAC8F11.03	SPCC663.01c
31	SPBC216.06c	SPAC23C11.08	SPAC13A11.04c	SPBC21C3.02c
32	SPCC1450.05c	SPBC3B8.02	SPAC1952.07	SPAC1F7.09c
33	SPAC22A12.16	SPBC17D1.06	SPAC14C4.03	SPBC365.11
34	SPAC12B10.07	SPAC11E3.06	SPAC19G12.06c	SPAC144.04c
35	SPAC4A8.05c	SPCC663.01c	SPAC2F7.04	SPCC4F11.03c
36	SPAC1B9.02c	SPAC167.04	SPAC1006.09	SPAC4G8.03c
37	SPAC16C9.05	SPAC14C4.12c	SPAC17G6.04c	SPBP8B7.06
38	SPAC23H3.08c	SPBC2A9.06c	SPAC22H12.02	SPCC1223.05c
39	SPAC2F7.04	SPAC24H6.13	SPBC725.02	SPAC1556.05c
40	SPAC22H12.02	SPAPB1A10.03	SPAC140.01	SPBC16C6.03c
41	SPBC725.02	SPAC12G12.03	SPAC1805.04	SPBC2G2.07c
42	SPAC21E11.03c	SPCC663.03	SPBC1709.10c	SPAC31G5.12c
43	SPBC12C2.02c	SPBC1539.10	SPBC577.15c	SPAC4G9.15
44	SPBC21B10.13c	SPCC1223.05c	SPAC1420.03	SPAC25B8.05
45	SPAC1687.13c	SPCC31H12.04c	SPBC29A10.03c	SPAC1071.07c
46	SPBC3F6.05	SPCC1739.08c	SPAC4F10.04	
47	SPBC4B4.03	SPAC2C4.07c	SPAPB1E7.12	
48	SPAC222.16c	SPAC1142.08	SPCC320.08	
49	SPAC4F10.04		SPCC830.07c	
50	SPAC19B12.10		SPAC4G9.05	
51	SPBC2G2.02		SPBC13G1.14c	
52	SPAC1834.04		SPCC24B10.08c	
53	SPAPB1E7.12		SPAC4H3.03c	
54	SPCC320.08		SPAC1805.05	
55	SPCC830.07c			
56	SPAC4G9.05			
57	SPBC13G1.14c			

**Figure 3.**
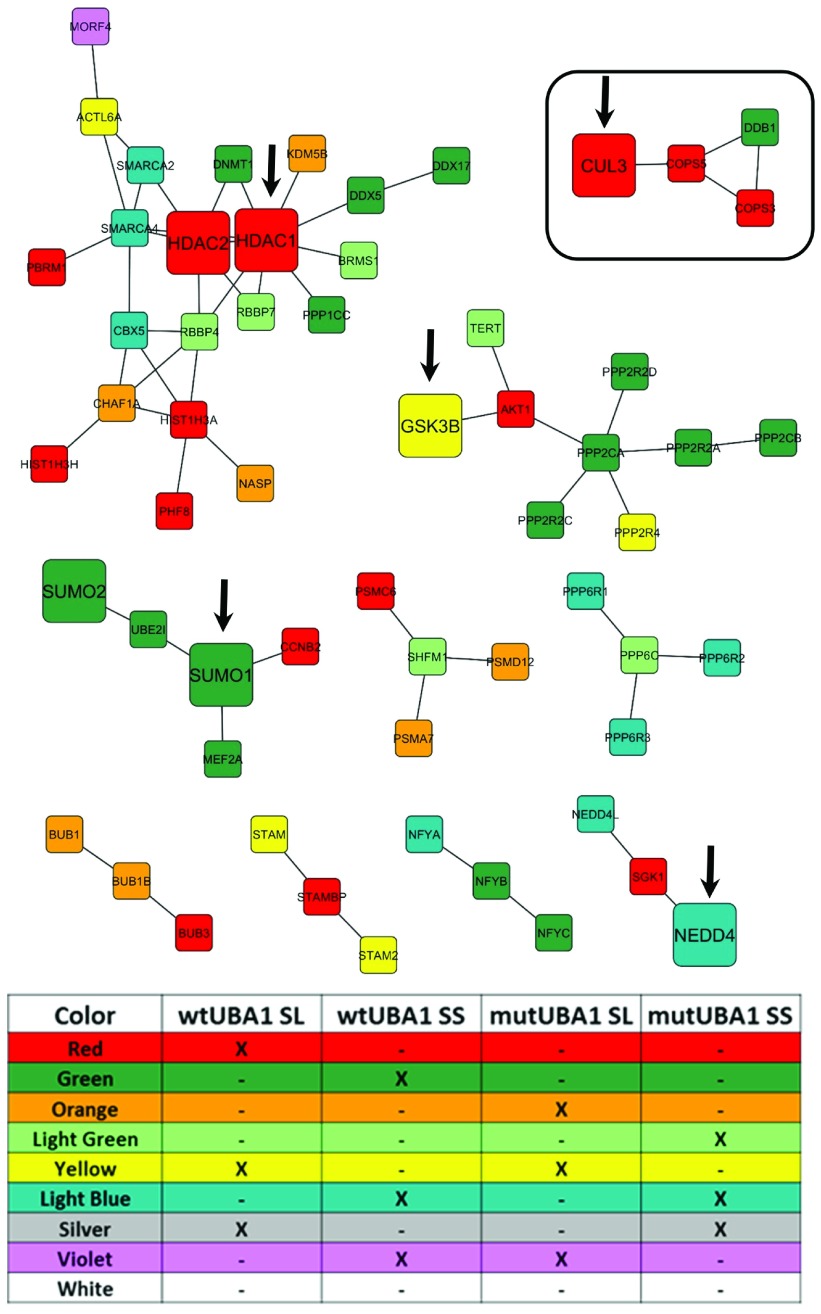
Human *UBA1* primary gene interaction network. Human orthologs of yeast genes identified from four independent SGA screens were used to map the corresponding protein-protein interaction network. Only proteins that have experimentally verified data (STRING) supporting a physical interaction with at least two additional genes/proteins identified in the *UBA1*-SGA screens are displayed. Of particular interest is the discovery of NEDD4, CUL3, GSK3, SUMO and HDAC in distinct network clusters. Proteins of interest are highlighted by a larger node size and indicated with an arrow.

**Figure 4.**
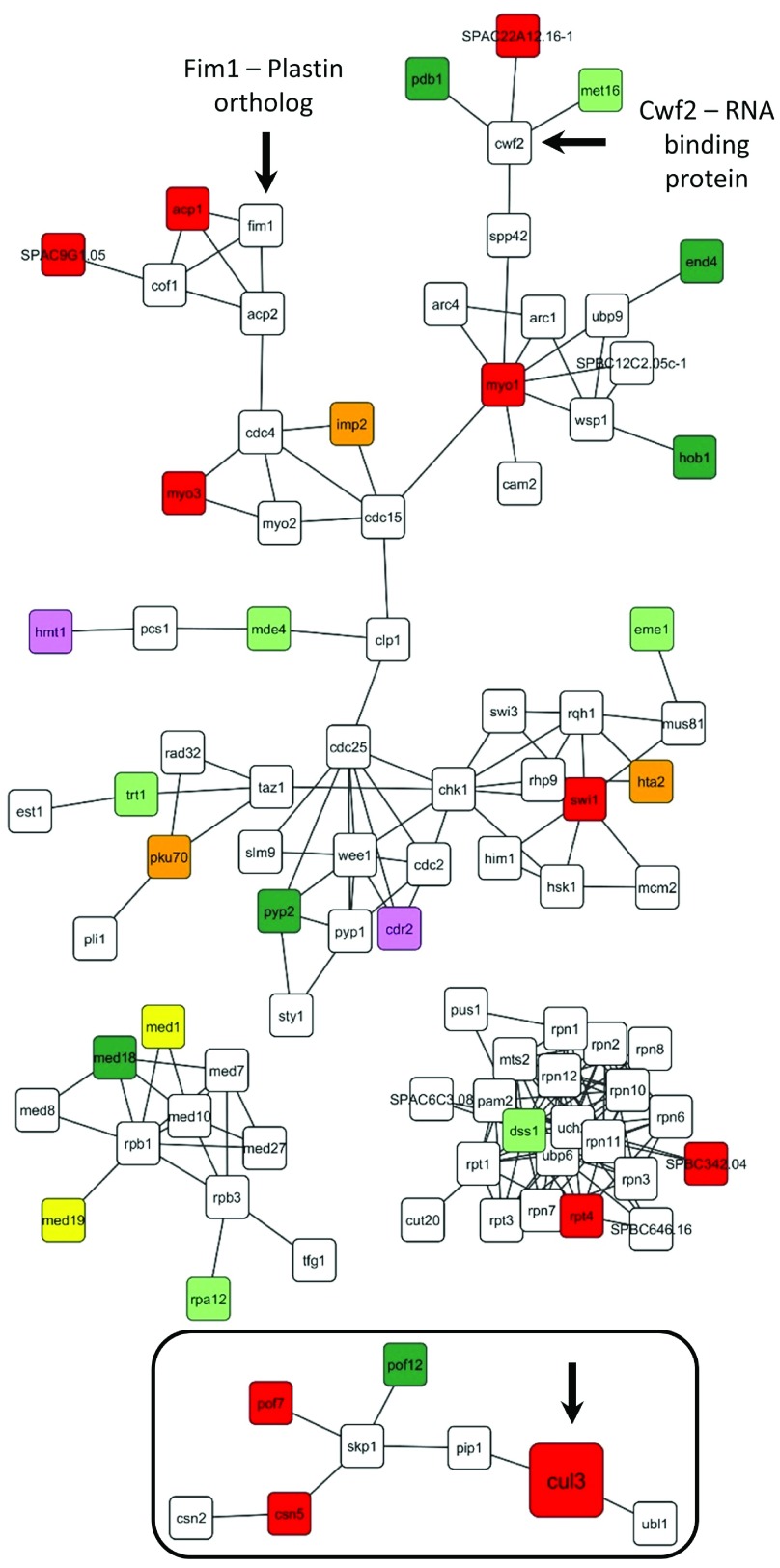
*S. pombe* extended *UBA1* gene interaction network (extracted from Figure S1 on the basis of identifying new primary hit interactions through a 1° neighbor). Primary yeast gene hits from all four SGA screens were combined and mapped based on highest confidence experimentally confirmed protein-protein interaction data. For this extended analysis, we also included first-degree neighbors of modifiers that were not directly identified in the SGA screens. Only selected gene clusters are shown here; the complete extended network is shown in Figure S1. When extrapolated to human, the yeast extended network analysis identified Csn5 and Cul3, two modifiers also identified in our human *UBA1* primary gene interaction network (boxed; see also Figure 3).

**Figure S1.**
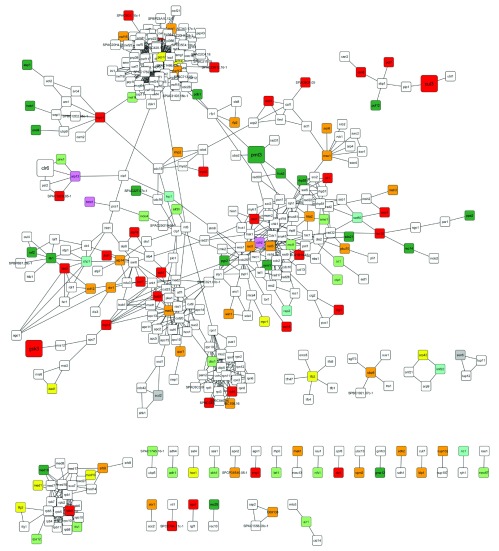
*S. pombe* extended *UBA1* interaction network (complete). The full protein-protein interaction network including first-degree neighbors for all fission yeast gene modifiers identified from our two independent SGA screens.

**Figure S2.**
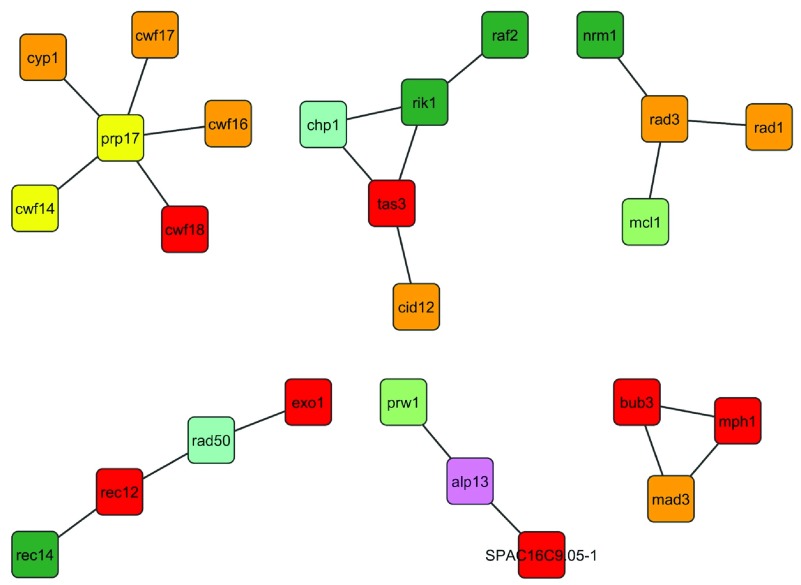
*S. pombe UBA1* primary gene interaction network. Fission yeast epistatic modifiers from all four SGA datasets were combined and used to map a protein-protein interaction network using the highest confidence protein interaction data (STRING-db).

**Figure S3.**
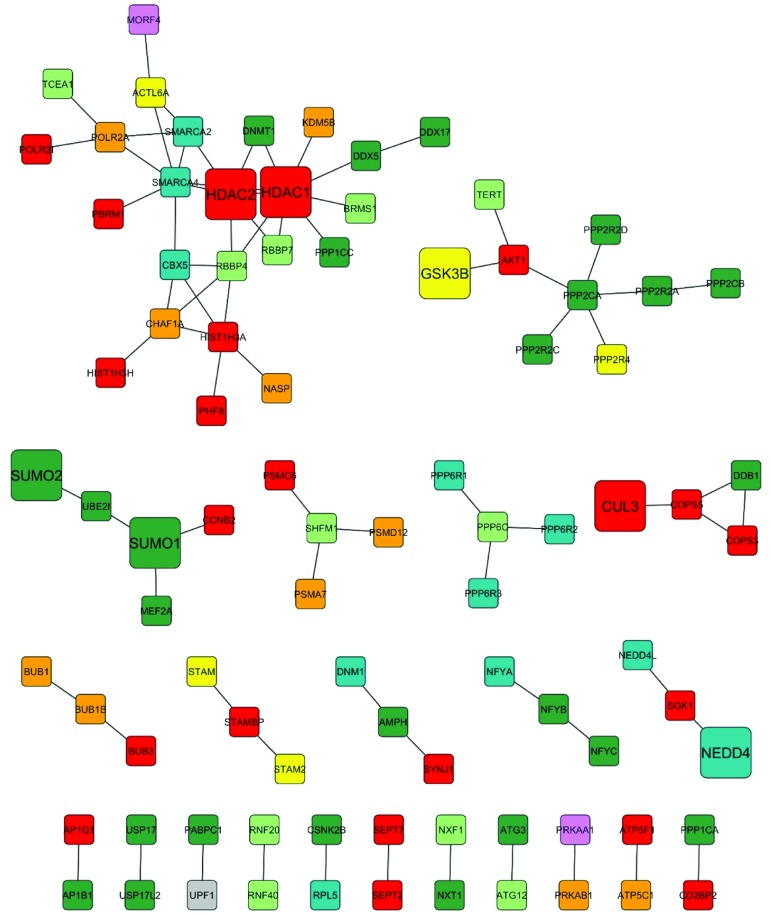
Complete human *UBA1* primary gene interaction network representing 22 clusters. Human orthologs of yeast genes identified from four independent SGA screens were used to map the corresponding protein-protein interaction network.

To generate our candidate human disease gene networks (that we shall call network clusters) we relied on two complementary approaches. Both approaches rely on networks constructed from published protein-protein interaction data from either human ( Figure 3 and Figure S3) or fission yeast ( Figure 4, Figure S1 and Figure S2). By including two types of genetic interactions, synthetic lethals or synthetic suppressors, yeast augmented network analysis (YANA), yields the most complete unbiased list of potential modifiers. In a first approach, we converted all 145 *S. pombe* modifier genes to their corresponding human orthologs. All the data were combined to create network cluster diagrams. Individual datasets were delineated using a color key. (Note that we designate each interaction as a synthetic lethal or synthetic suppressor *a priori*, and include them both as it is difficult to predict which of these classes will have potential therapeutic value). Using the highest confidence (0.9) experimentally confirmed protein-protein interaction data (from www.string-db.org), we used cytoscape to draw network cluster modules that include all modifier genes. We identified 22 clusters using this approach ( Figure S3). We then limited the number of networks by focusing on those modifiers that shared at least two interactions with other modifiers shrinking the number of clusters to 10 ( Figure 3). Within our human primary network clusters, we identified several genes that represent compelling therapeutic targets. These included GSK3 (Glycogen Synthase Kinase-3), CUL3 (Cullin-3) and NEDD4 (E3 ubiquitin ligase) along with SUMO1 (Small Ubiquitin Modifier-1) and HDAC (Histon deacetylase), a known chromatin modifier. Interestingly, GSK3 inhibitors increase SMN levels in spinal muscular atrophy patient-derived fibroblasts and mouse motor neurons ^28^. The HDAC inhibitor Trichostatin A increases SMN expression and survival in a mouse model of spinal muscular atrophy ^29^. Therefore, evidence already exists that genes within our clusters affect SMA biology.

In our second approach, we constructed networks from just the *S. pombe* modifier genes using high confidence *S. pombe* protein-protein interaction data from STRING. For this analysis, we identified network clusters that were expanded to include all the so-called “first-degree (1°) interacting proteins”, proteins not identified in our SGA screens but that physically interact with a genetically identified modifier ( Figure S1). The rationale underlying our approach to generate larger clusters comes from the facts that the 1° neighbor may be an essential gene (and therefore it is not present in our deletion library) or may fail to be detected in our screens for other, unknown reasons. This “Cluster with 1° neighbor” analysis reveals new interactions not observed in the yeast primary *UBA1* gene interaction network ( Figure S2). A few examples of these types of clusters are shown in Figure 4. The clusters include several genes all of which were uniquely identified in the wildtype screen, including both synthetic lethals and synthetic suppressors. Of particular interest is the cluster of proteins surrounding Skp1(S-phase kinase associated protein-1), a protein that interacts and stablilizes F-box proteins, additional F-box proteins, and proteins involved in cullin deneddylation and neddylation. In addition, we found that the RNA-binding protein Cwf2 interacts directly with three of our modifiers, and the plastin ortholog Fim1, interacts with two. Interestingly, Plastin 3 (PLS3) has been identified as a disease modifier in animal models of spinal muscular atrophy ^30– 32^.

We then identified correlations between the extended *S. pombe* network clusters ( Figure 4) and the human primary network clusters ( Figure 3). In both networks, we identified components of the COPS complex, a known regulator of E3 cullins, and the E3 cullin Cul3. Note that Cul3 was not present in our primary *S. pombe* network ( Figure S2) yet was present in the more extended network clusters that incorporate 1° neighbors; this illustrates the importance of adding 1° neighbors to our analysis of the *S. pombe* data.

**Figure 5.**
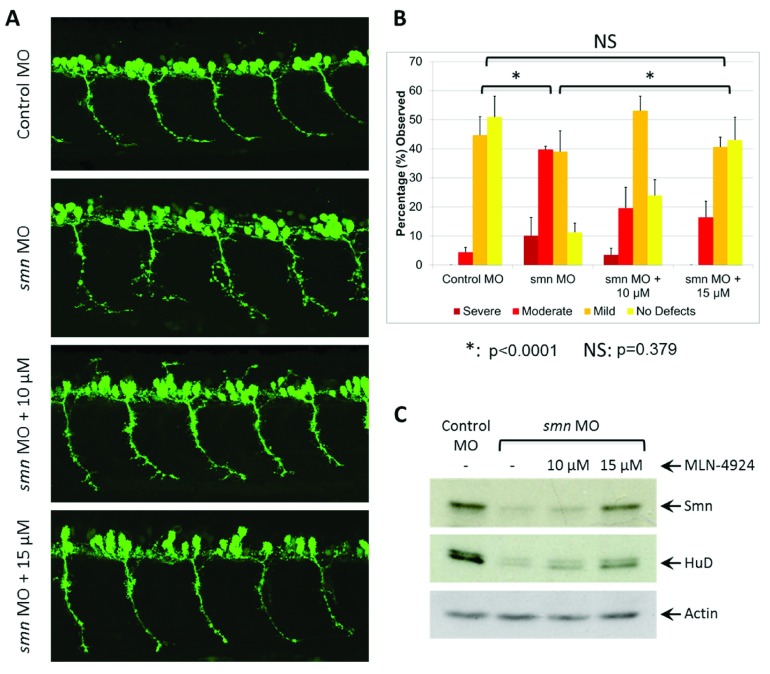
MLN4294 rescues the abnormal neuron outgrowth in Smn-depleted zebrafish. ( **A**) Representative lateral views of motor axons in *Tg*( *mnx1:GFP*) zebrafish embryos expressing GFP in motor neurons and injected with control MO and then grown in 10 or 15 µM MLN4294. ( **B**) Quantification of the effects of MLN4294 on motor axon development in zebrafish. Motor axons were scored in *Tg*( *mnx1:GFP*) embryos injected with control MO, and subsequently (10 hrs post-injection) incubated in 10 or 15 µM MLN4294. Embryos were classified as severe, moderate, mild, or no defects based on the severity of motor axon defects, and the percentage of each group is shown. Data in all graphs are represented as mean and SEM. ( **C**) Western blot analysis of Smn and HuD protein following treatment with MLN4294. Quantification of the results is shown in Figure S5.

**Figure 6.**
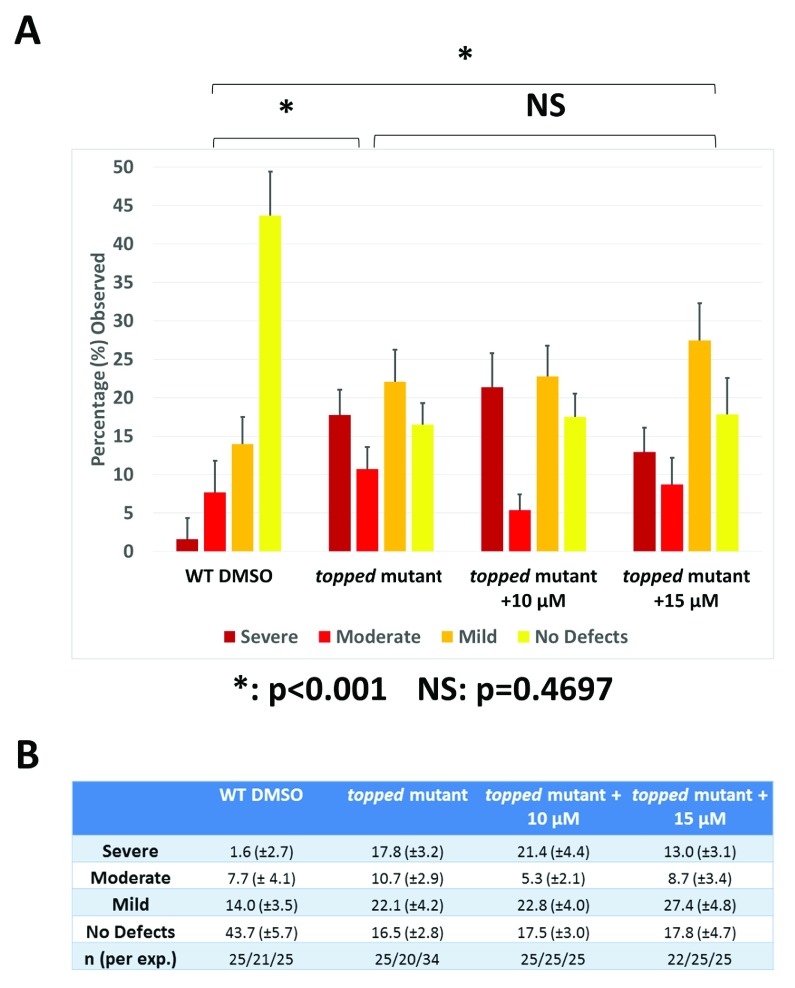
Treatment of zebrafish with MLN4294 fails to rescue defects in ventral motor axon guidance in the *topped* mutant. ( **A**) Quantification of the effects of MLN4294 on motor axon guidance in the zebrafish *topped* mutant. Motor axons were scored in embryos injected with DMSO, 10 or 15 µM MLN4294. Embryos were classified as severe, moderate, mild, or no defects based on the severity of motor axon defects, and the percentage of each group is shown. Data in all graphs are represented as mean and SEM. ( **B**) Experimental data.

Together, these data suggest a prominent role for E3 ubiquitin ligases in modulating UBA1 and potentially SMN1, making Cul3 the primary target for further analysis. The E3 ubiquitin ligase might inhibit SMN function, as ubiquitination often leads to protein degradation. If so, we reasoned that inhibition of E3 ubiquitin ligase might *enhance* SMN activity and thus suppress loss of function mutations in SMN1. To directly test the hypothesis that inhibition of E3 ubiquitin ligases can suppress a SMN1-spinal muscular atrophy phenotype, we turned to a vertebrate model of SMN deficiency previously reported in zebrafish ^33^. Knockdown of Smn in zebrafish embryos causes developmental defects in motor neuron axonal outgrowth that include truncations and abnormal branching of neurons ^24^. Motor neuron axon defects can be corrected by injection of mRNAs encoding wildtype human SMN ^25^. We first confirmed that injection of *smn* morpholino (MO) into wildtype zebrafish causes severe motor axon abnormalities as compared to control uninjected embryos ( Figure 5A and 5B). We then tested whether an inhibitor of E3 ubiquitin ligases (MLN4294) would suppress the motor axon abnormalities. Addition of the Nedd8-E1 activating enzyme inhibitor MLN4294 (that blocks cullin-RING E3 ligases), at concentrations ranging from 10 µM to 15 µM, caused a concentration-dependent reduction in the degree of abnormal motor axon branching ( Figure 5A and 5B). At 15 µM inhibitor we found motor axon abnormalities that were completely suppressed: defects were not significantly different to those observed in uninjected embryos (P=0.379). At higher concentrations of drug (20 µM), the rescue was less pronounced ( Table 2) probably because higher levels of drug led to defects in development. Thus, E3 ligase inhibition can rescue neuronal defects caused by Smn protein depletion in zebrafish. In control experiments, MLN4294 failed to rescue the zebrafish mutant *topped* ( Figure 6), a mutant defective in neuronal axon guidance ^34^. We also tested a drug that inhibits sumolyation corresponding to an unrelated target (SUMO) identified in our human primary network clusters ( Figure 3); addition of this compound had no effect on the spinal muscular atrophy model ( Figure S6) necessarily surprising since *pmt3*, which encodes the yeast ortholog of *SUMO1*, has the opposite effect of *cul3* when deleted. Having been identified as a synthetic suppressor rather than a synthetic lethal, perhaps compounds that activate rather than inhibit sumolyation would be beneficial as a therapeutic.

**Table 2.** Complete dataset for zebrafish experiments.

	Control MO	smn MO	smn MO + 10 µM	smn MO + 15 µM	smn MO + 20 µM
**Severe**	0 (± 0)	10.1 (± 6.3)	3.5 (± 2.3)	0 (± 0)	2.8 (± 2.4)
**Moderate**	4.4 (± 1.7)	39.7 (± 1.2)	19.6 (± 7.2)	16.4 (± 5.6)	11.3 (± 7.1)
**Mild**	44.7 (± 6.4)	39 (± 7.2)	53.1 (± 5)	40.6 (± 3.4)	46.8 (± 9.6)
**No Defects**	50.9 (± 7.2)	11.2 (± 3.2)	23.9 (± 5.5)	43 (± 7.9)	39.1 (± 19)
**n (per exp)**	23/19/27/25	23/20/28/27	21/23/27/26	19/17/26/26	22/25/26

**Figure S5.**
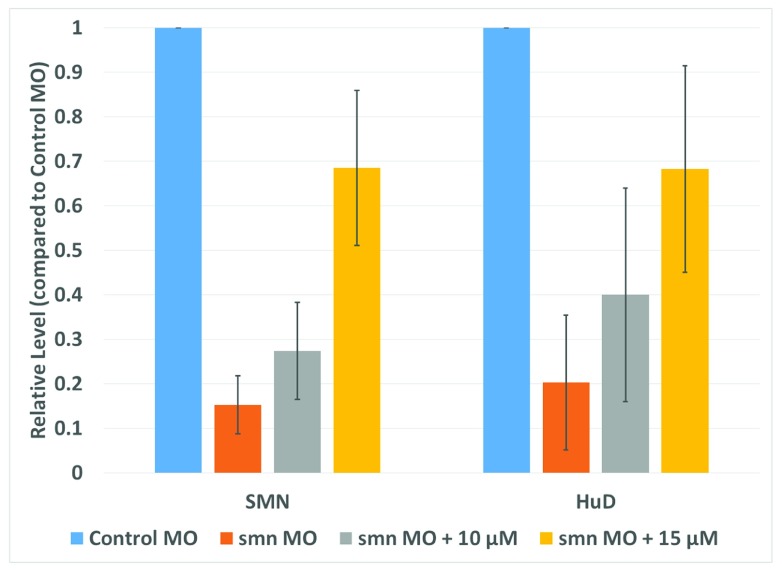
Expression of Smn and HuD in zebrafish embryos. Quantification of western blot results shown in Figure 5C. Treatment of zebrafish embryos with MLN4294 results in approximately a 3-fold increase in both Smn and HuD levels 18 hrs following treatment.

**Figure S6.**
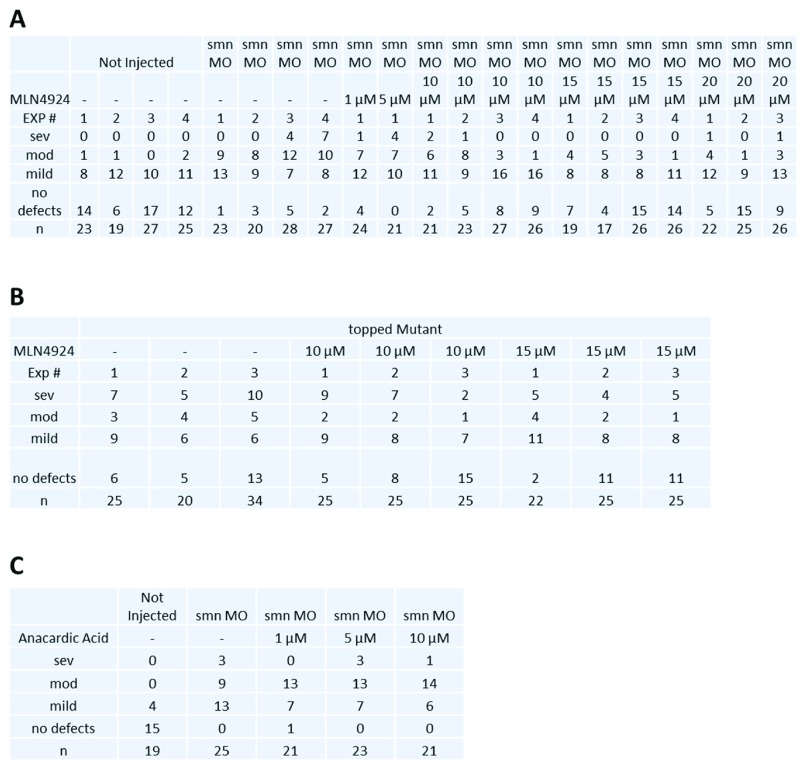
Zebrafish raw data. **A**) MLN4924 treatment in smn MO zebrafish. **B**) MLN4924 treatment in topped mutant zebrafish. **C**) Anacardic acid treatment in smn MO zebrafish.

To determine how E3 ubiquitin ligases might influence Smn1 function, we examined the protein levels for both Smn and HuD. HuD is a known SMN interacting protein that binds RNAs controlling their translation and stability and functions in neural development and plasticity ^34– 36^. We observed greater than a 2-fold increase in both Smn and Hud protein levels following treatment with MLN4294, a NEDD8 activating enzyme (NAE) inhibitor that prevents activation of E3 ligases ( Figure 5C and Figure S5). It is therefore possible that rescue of the neuronal defects in our zebrafish model might involve stabilization of Smn as well as of additional proteins within the Smn network.

Growth rate data of expression of UBA1 in S. pombe UBA1 was expressed in S. pombe under inducing (- thiamine) and non-inducing conditions (+ thiamine) in wildtype strains (wtUBA1) and the disease-causing variant wildtype 1617UBA1 (mutUBA1), under the control of a regulatable nmt1 promoter. Growth rate data of expression of UBA1 under inducing (- thiamine) and non-inducing conditions (+ thiamine) in wtUBA1 and mutUBA1 are shown. For each condition there are six replicates.

## Discussion

In this paper we introduce YANA, a fission yeast genetic assay that that when applied to a specific human disease gene can leverage protein-protein interaction data to characterize human disease networks. From these networks, we can identify and prioritize genetic pathways likely to modify the disease-associated gene activity, and predict those genes that can be exploited as therapeutic targets. Using *UBA1*, a gene associated with X-linked spinal muscular atrophy (XL-SMA), we found a high degree of homology between our yeast modifiers and their corresponding human genes (>80%), underscoring the extensibility of the assay. Specifically, we identified several network clusters and high priority targets for therapeutic intervention, including GSK3 and HDAC both of which are potential therapeutic targets for SMA. In addition, we found two novel related targets Cul3 (a cullin required for E3 ubiquitin ligase activity) and NEDD4, an E3 ubiquitin ligase.

Our yeast system, with its small but complex eukaryotic genome, and complete deletion library, is unique in allowing unbiased genome-wide screening of deletions that alter human disease gene activity. Moreover, YANA can be applied to any human gene, regardless of the phenotype or availability of endogenous mutations. The number of candidate genes identified by YANA for *UBA1* represents a fraction of the approximately 30,000 genes in the human genome, providing a significant enrichment of potential modifiers. Therefore, YANA offers a simple, cost-effective, and relatively rapid technology that could be applied to all human genetic diseases.

## Data availability

F1000Research: Dataset 1. Growth rate data of expression of UBA1 in *S. pombe*, 10.5256/f1000research.4188.d28505 ^37^.

## Figures and Tables

**Figure S4.**
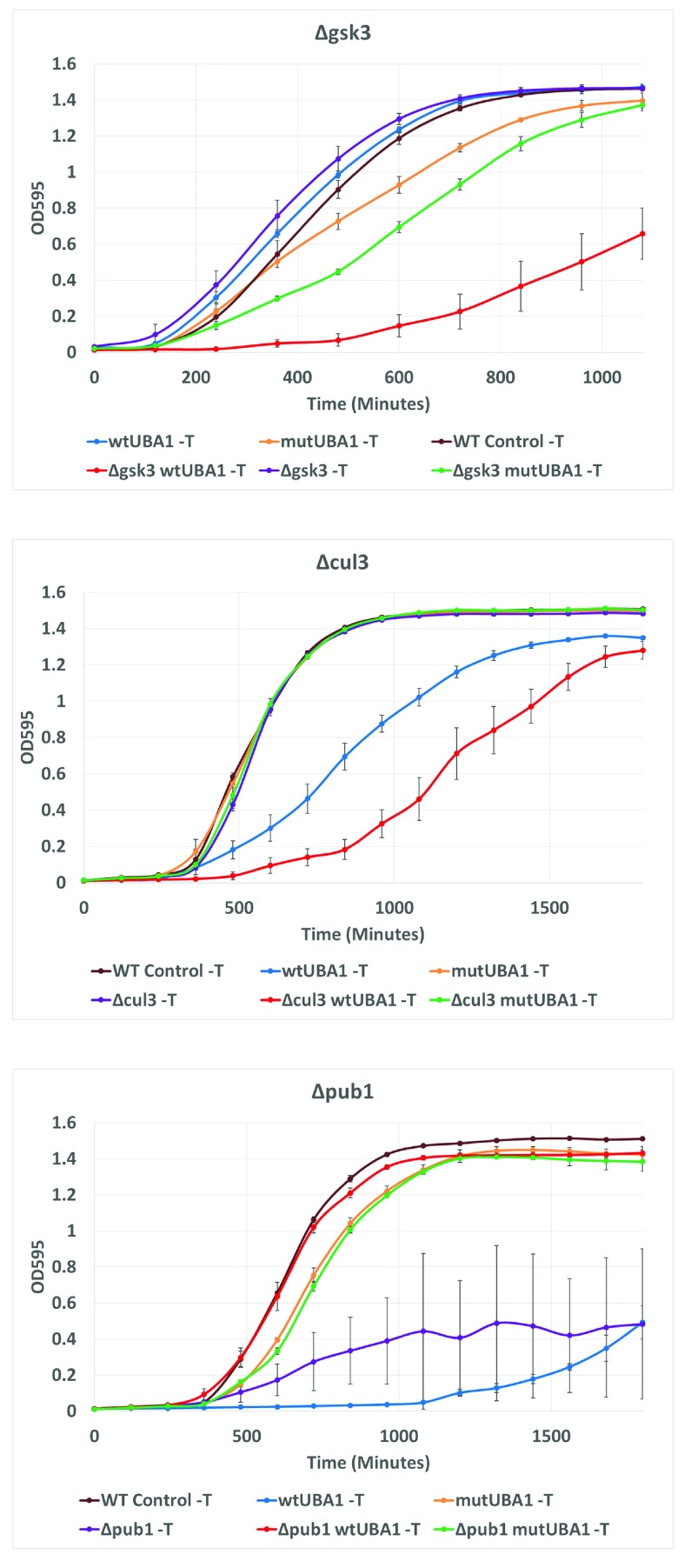
Validation of SGA data for *cul3*, *pub1* and *gsk3*. Growth curves of the 3 double mutants confirmed our original SGA data showing that the deletions of *cul3* and *gsk3* are synthetically lethal with OE of *UBA1*, and the deletion of *pub1* ( *NEDD4*) suppresses the toxicity associated with OE of wildtype *UBA1*. For *gsk3* and *cul3*, the cells were washed to remove thiamine and immediately placed in the plate reader whilst for *pub1*, cells were first pre-induced for 22 hrs. Deletions of *cul3*, *pub1*, and *gsk3* were confirmed by PCR.
