# Supplementary material for: Yeast Augmented Network Analysis (YANA): a new systems approach to identify therapeutic targets for human genetic diseases
Source: F1000Res. 2014 Jun 2;3:121. [Version 1] doi: 10.12688/f1000research.4188.1 (PMC4097366; doi:10.12688/f1000research.4188.1)
Supplement: Peer review history

# Peer review: Referee response for version 1

**DOI:** 10.5256/f1000research.4484.r5211

**Published:** 2014-06-23

**Authors:** Jasper Rine

**Affiliations:** 1 Department of Molecular and Cell Biology, University of California Berkeley, Berkeley, CA, USA

The Wiley *et al *manuscript describes a beautiful synthesis of contemporary genetic approaches to, with astonishing efficiency, identify lead compounds for therapeutic approaches to a serious human disease. I believe the importance of this paper stems from the applicability of the approach to the several thousand of rare human disease genes that Next-Gen sequencing will uncover in the next few years and the challenge we will have in figuring out the function of these genes and their resulting defects. This work presents a paradigm that can be broadly and usefully applied.

 In detail, the authors begin with gene responsible for X-linked spinal muscular atrophy and express both the wild-type version of that human gene as well as a mutant form of that gene in *S. pombe*.  The conceptual leap here is that progress in genetics is driven by phenotype, and this approach involving a yeast with no spine or muscles to atrophy is nevertheless and N-dimensional detector of phenotype.

The study is not without a small measure of luck in that expression of the wild-type *UBA1* gene caused a slow growth phenotype which the mutant did not.  Hence there was something in *S. pombe* that could feel the impact of this protein. Given this phenotype, the authors then went to work and using the power of the synthetic genetic array approach pioneered by Boone and colleagues made a systematic set of double mutants combining the human expressed UBA1 gene with knockout alleles of a plurality of *S. pombe* genes.  They found well over a hundred mutations that either enhanced or suppressed the growth defect of the cells expressing UBI1.  Most of these have human orthologs.  My hunch is that many human genes expressed in yeast will have some comparably exploitable phenotype, and time will tell.

Building on the interaction networks of *S. pombe* genes already established, augmenting these networks by the protein interaction networks from yeast and from human proteome studies involving these genes, and from the structure of the emerging networks, the authors deduced that an E3 ligase modulated *UBA1* and made the leap that it therefore might also impact X-linked Spinal Muscular Atrophy.

Here, the awesome power of the model organism community comes into the picture as there is a zebrafish model of spinal muscular atrophy. The principle of phenologs articulated by the Marcotte group inspire the recognition of the transitive logic of how phenotypes in one organism relate to phenotypes in another.   With this zebrafish model, they were able to confirm that an inhibitor of E3 ligases and of the Nedd8-E1 activating suppressed the motor axon anomalies, as predicted by the effect of mutations in *S. pombe* on the phenotypes of the UBA1 overexpression.

I believe this is an important paper to teach in intro graduate courses as it illustrates beautifully how important it is to know about and embrace the many new sources of systematic genetic information and apply them broadly.

I have read this submission. I believe that I have an appropriate level of expertise to confirm that it is of an acceptable scientific standard.

# Peer review: Referee response for version 1

**DOI:** 10.5256/f1000research.4484.r4970

**Published:** 2014-06-05

**Authors:** Corey Nislow

**Affiliations:** 1 Department of Pharmaceutical Sciences, University of British Columbia, Vancouver, BC, Canada

This paper addresses the vexing problem of how to unravel the complexity of human diseases. Such diseases often involve interactions between genes, and the question is therefore well-suited to an automated genetic approach, such as Synthetic Genetic Array technology, which was pioneered in bakers yeast and more recently adopted to another well-characterized model system, namely *S. pombe.* In this study the authors examine conserved genetic interactions between a fission yeast genes and a human query gene (both wildtype and loss-of-function mutants) then combine their experimental data with bioinformatics to characterize the network of genetic interactions and protein-protein interactions. They then leverage this network to identify interacting partners that comprise novel therapeutic targets.

The example presented in this paper is the human *UBA1* gene which encodes a gene that, when mutated, results in human X-linked spinal muscular atrophy disease. They perform whole genome genetic interaction mapping for digenetic interactions with both wild type and mutant versions of the gene, build a network with the observed interactions, and then expand that primary network by adding the known partners that interact with members of the primary network. They then turned to the zebrafish vertebrate model system to validate their newly identified therapeutic targets.

This study adds another tool to the armamentarium of systems biology approaches with which the complexity of human diseases can be dissected. Because the system is new in the sense that fission yeast has been largely unexplored (compared to its budding yeast relative from which it has diverged by several hundred million years) the observations on fission yeast, when combined with those from other model systems, provide an attractive complementary approach to understanding complex human biology.

Given that this study builds on earlier approaches, the authors should cite the previously published studies on genetic interactions, chemical genetic interactions and protein-protein interactions using budding yeast, fly and other models. Similarly, the comprehensive gene expression profiling approaches used in cultured mammalian cells as well as more recent whole genome RNA interference and CRISPR-based disruption screens should be cited.

From a methodological perspective, in the interaction maps presented in Figures 3 and 4 it would be useful to describe or cite the methods used to define the links between genes. In the Results, where the authors describe the results of the different screens which include wild type and mutant human protein interactions as well as primary and secondary interactions it would be instructive to provide a graphic or flow chart that depicts how all the key pieces of data from each independent screen in fission yeast (and their analysis) were collapsed and presented together. It would also be instructive to provide background information or appropriate citations on the two compounds used in the zebrafish experiments, specifically their known or suspected mechanisms of action as well as the data to support these mechanisms.

In the Discussion, the authors have room to put the YANA screening platform into context, comparing it to other orthologous genome-wide approaches.

A key innovation in this study arises from the unique approach used in analyzing the data. Specifically, the conserved yeast genes that modify the phenotype induced by expressing a human gene of interest are assembled into human disease gene networks which are then augmented by additional protein-protein interaction data. Augmenting traditional network analysis in this manner may permit the identification of new therapeutic targets and target pathways. In summary, this comprehensive, genome-wide work represents an important addition to the systems biology toolkit to understand the complexities of human biology and the impact of this and subsequent data sets will surely increase as additional screens are added to the compendium.

Minor points

 

- Western blot and growth curve: *"or eight generations"* is a typo, also the type of media the cells were grown in *“exponentially for 16 hours”* should be listed.
- In Figure 2 it is not clear which band the tubulin arrow is pointing to- the loading control bands should be indicated directly on the blot/gel. Also, it is apparent from this loading control that the amount of UPA1 expressed is extremely high, the authors should comment on the fact that the overexpressing lanes are actually UNDERloaded relative to the non-expressing lanes.
- Third paragraph of the Results: the software programme cytoscape should include a reference.
- Third paragraph of the Results: histone is misspelled " *histon*".
- Sixth paragraph of the Results: the authors should clearly distinguish between the published data in zebrafish and their new data collected in this study
- Sixth paragraph of the Results: as mentioned be inhibitor’s mechanism of action should be described in more detail
- Second paragraph of the discussion: approximately 30,000 genes in the human genome should be cited and probably corrected to the conventional number of 21,000.

I have read this submission. I believe that I have an appropriate level of expertise to confirm that it is of an acceptable scientific standard.
